# Rearing Host Dependency of Ovariole Number and Body Size in *Campoletis chlorideae* Uchida (Hymenoptera: Ichneumonidae)

**DOI:** 10.3390/insects14050483

**Published:** 2023-05-20

**Authors:** Liangheng Tian, Xiongya Wang, Yu Wang, Xiaohang Gu, Xin Li, Shiheng An, Xinming Yin, Sufen Bai

**Affiliations:** 1 Henan International Laboratory for Green Pest Control, Henan Engineering Laboratory of Pest Biological Control, College of Plant Protection, Henan Agricultural University, Zhengzhou 450002, China; 2 Institute of Biology Co., Ltd., Henan Academy of Sciences, Zhengzhou 450008, China

**Keywords:** *Campoletis chlorideae*, ovariole, body size, host species, instar

## Abstract

**Simple Summary:**

*Campoletis chlorideae*, a solitary larval endoparasitoid, has a wide host range. It has great biocontrol potential against some major worldwide agricultural economic pests, which is valuable and shows potential for development. The ovariole is where the eggs develop and mature. Ovariole number variability is species-specific, and closely related to fecundity. However, the ovariole characteristics of the parasitoid, particularly the effect of rearing host on ovariole number and body size, as well as their correlation, have not been studied until now. Our study shows that the parasitoid has paired ovaries which contain multiple ovarioles, and the ovariole number displays great individual variability and asymmetry between bilateral ovaries. Moreover, suitable host species and larval instars could improve their ovariole number and body size. There is a strong positive correlation between ovariole number and body size, namely that larger body size represents more ovarioles. Overall, our findings indicate that the parasitoid has great reproductive plasticity by regulating ovariole number. Additionally, body size combined with ovariole number can be used as an important index to evaluate the quality of the parasitoid. Our works provide practical help for the development and application of biocontrol using the parasitoid.

**Abstract:**

*Campoletis chlorideae* has great biocontrol potential against some major noctuid pests. In order to achieve the commercial development and utilization of *C. chlorideae*, this study focused on the effect of rearing host species and larval instars on the ovariole number and body size of this wasp. Firstly, the morphology of the reproductive system and ovarioles of female wasps were observed. The number of ovarioles displayed great variability and asymmetry between bilateral ovaries. Moreover, the effect of four host species on ovariole number and body size of *C. chlorideae* were studied. The wasps had a larger ovariole number and body size when reared in *Helicoverpa armigera*. Additionally, the ovariole number and body size were larger when reared in the third instar larvae than in the first or second instar larvae of *H. armigera*. There was a strong positive correlation between the ovariole number and body size of *C. chlorideae*. The ovariole number and body size of the wasp could be improved under optimized artificial rearing conditions. According to these results, body size combined with ovariole number can be used as an important index to evaluate the quality of *C. chlorideae*. This study provides important clues for the development and application of biocontrol using *C. chlorideae*.

## 1. Introduction

The ichneumonid *Campoletis chlorideae* Uchida (Hymenoptera: Ichneumonidae), a solitary larval endoparasitoid of over 30 lepidopteran pests, can parasitize many major agricultural pests including *Helicoverpa armigera* Hübner, *H. assulta* Guenée, *Mythimna separata* Walker, *Spodoptera frugiperda* JE Smith, and *S. litura* Fabricius (all Lepidoptera: Noctuidae), etc. [1,2,3,4,5,6,7]. It has significantly reduced the *H. armigera* populations in many parts of the world [3,6,8], with a parasitism rate up to 80% in different ecosystems [8,9]. It is worth noting that *S. frugiperda*, a new invasive pest that has caused significant damage to crops in many countries and regions, could be parasitized by *C. chlorideae* [1,2]. Because of its high parasitic efficiency, *C. chlorideae* has been recognized as an effective biological control agent against many major agricultural noctuid pests on various crops including cotton, tomato, peanut, tobacco, and cabbage [3,5,6]. However, there are still many problems with the mass rearing of *C. chlorideae*.

It is crucial to understand the reproductive characteristics of parasitoids for utilizing the biological control agents to control pests. In general, the ability of parasitic wasps to control pests is determined by their fecundity or egg-laying capacity. As we all know, eggs are produced in the ovarioles, so the study of the morphology, type, and number of ovariole is necessary to help improve the fecundity of parasitoids. For social wasps, the different morphometric characteristics of ovarioles show different reproductive status between castes, with the queen wasps have the greatest degree of ovarian development [10]. Knowing the type of ovarioles is helpful to the cognition of oogenesis. Parasitic wasps have meroistic polytrophic type ovarioles which contain sequentially arranged oocytes accompanying nurse cells. Knowledge about ovigeny in parasitoids is important for success in biological control applications [11,12]. Therefore, it is very important to make clear the morphology and type of ovariole of *C. chlorideae*.

Ovarioles number is a very important indicator of fecundity. More ovarioles mean higher egg production or fecundity [13,14]. The number of ovarioles per ovary is species-specific. Some species have a fixed number. For examples, *Apanteles plutellae* Kurdjumov (Hymenoptera: Braconidae) and *Diadromus collaris* Gravenhorst (Hymenoptera: Ichneumonidae), respectively, have 4 and 6 ovarioles in their ovaries [15]. Some species have intraspecific variation in ovariole number which is affected by biotic or abiotic factors. For endoparasitoids, the influence of the host is the most prominent among the biotic factors, including host species and host instars. We hold the opinion that the species with variational ovariole number have great reproductive plasticity.

Body size is also an important indicator of fecundity for parasitic wasps. Usually, there is positive correlation between body size and ovariole number for the wasps with variational ovariole number [13,16]. For example, the largest female, with 26 ovarioles, had 1.6 times as many ovarioles as the smallest female, with 16 ovarioles in endoparasitoid *Venturia canescens* Gravenhorst (Hymenoptera: Ichneumonidae) [13]. Similar differences were observed in *Encarsia formosa* Gahan (Hymenoptera: Aphelinidae) (five to sixteen ovarioles) [17] and *Bracon hebetor* Say (Hymenoptera: Braconidae) (one to nine ovarioles) [18]. In addition, egg production was also positively correlated with the body size of female adults. The larger females of *Agrothereutes lanceolatus* Walker (Hymenoptera: Ichneumonidae) [19], *Asobara tabida* Nees (Braconidae: Alysiinae) [20] and *Aphaereta genevensis* Fischer (Hymenoptera: Braconidae) [21] carried a greater number of eggs in their ovarioles. Females with larger body size have more resources to allocate, and therefore body size is positively correlated with reproductive traits such as ovariole number, egg size, initial egg load, lifetime fecundity, and life span [22]. Therefore, body size represents the quality of parasitoids, and the larger-bodied parasitoids have greater potential to control pests. How to produce a high quality biocontrol agent is an important goal of artificial rearing of parasitic wasps.

In view of the lack of research on the reproductive characteristics of *C. chlorideae*, this study focused on the correlation between the host and the female wasps’ ovariole number and body size. Firstly, the reproductive system structure of females was observed, and the morphological character and number variation of ovarioles were investigated. The effect of host species and instars on the ovarioles number and body size of wasp was studied to determine the optimal breeding host. Furthermore, the correlation between ovariole number and body size was verified. Our study helps to improve artificially reared *C. chlorideae* for high potential to control pests.

## 2. Materials and Methods

### 2.1. Culture of Parasitoid and Host Colonies

*H. armigera* were originally collected from the tobacco or pepper fields in Xuchang, China, in June 2019. Other lepidopteran species, *M. separata*, *S. litura* and *S. frugiperda*, were bought from Henan Jiyuan Baiyun Industrial Co., Ltd. Larvae of *H. armigera* and *S. litura* were maintained on an artificial diet cooked with soybean powder (80 g), yeast powder (30 g), wheat germ (150 g), sucrose (20 g), casein (40 g), ascorbic acid (3 g), compound vitamin (8 g), methyl parahydrobenzoate (3 g), sorbic acid (3 g), agar (20 g), formaldehyde (2 mL), acetic acid (4 mL), and distilled water (1500 mL), which referred to and modified the method described by Wang and Dong [23]. The artificial diets for larvae of *M. separata* and *S. frugiperda* were different but similar to *H. armigera*. The diet of *M. separata* contained extra added dried corn leaf powder, and the larvae of *S. frugiperda* were fed fresh corn leaves before the 3rd instar and fed the diet containing extra corn flour after the 3rd instar, and their adults were fed with 10% honey solution. Larvae and adults were kept in an artificial climatic box at 27 ± 1 °C, 60–80% RH and 14 L:10 D photoperiod (ZRG-250A-L, Shanghai Binglin Electronic Technology Limited Company, Shanghai, China). 

Cocoons of *C. chlorideae* were collected from a tobacco field in Xuchang, Henan Province, China in 2019. The species identification of *C. chlorideae* was performed by Dr. S. Bai. The 2nd instar larvae of the host *H. armigera* were used to rear *C. chlorideae.* Oviposition was performed by placing a mated female wasp and a host larva in a transparent glass vial (8.0 cm length × 1.5 cm diameter), and the host larva was removed immediately after a single oviposition [24]. The host larvae were discarded if accidentally parasitized more than once. The parasitized host individuals were kept separately in a clear plastic box (3 × 3 × 3 cm) with an artificial diet and placed in an artificial climatic box at 25 ± 1 °C, 70 ± 10% RH and a photoperiod of 14:10 h (L:D). After 6–7 days, the final instar larvae of wasps emerged from the host and were immediately pupated in a cocoon. Two days later, cocoons were collected in clear plastic boxes (17 × 6 × 6 cm) until wasps emerged. The adult wasps were fed with a 10% honey solution and kept at 17 ± 1 °C, 60–70% RH with a photoperiod of 14:10 h (L:D).

### 2.2. Dissection and Morphological Observation of Ovarioles

The mated females of *C. chlorideae* were 10% honey-fed and host-deprived at 17 ± 1 °C, and were dissected for morphological observation at 5–10 days after emergence. To count the number of ovarioles, dissection of each wasp was conducted following the method of Eliopoulos et al. [13]. Each wasp was killed by freezing and placed in insect phosphate-buffered saline (PBS), and then the wasp was dissected by grasping the thorax with forceps and pulling the long ovipositor distally with another pair of forceps. This enabled the ovaries and the rest of the reproductive system to be extracted. Paired ovaries were then placed in a drop of PBS solution on a microscope slide, and the ovarioles were teased apart with micropins for microscopic observation and photographing through a Canon Power shot A650 IS under a Leica inverted phase contrast microscope (SZ2-LGB, Olympus, Tokyo, Japan).

### 2.3. Statistic of Ovarioles

To investigate the variation of ovariole numbers in paired ovaries of *C. chlorideae*, the ovariole numbers of ovaries were counted, respectively, in 230 female wasps aged 0–10 days. The frequency distribution of ovariole number in unilateral ovaries and total ovaries were statistically analyzed. Meanwhile, the ovariole numbers of bilateral ovaries were subtracted to show the symmetry of ovariole numbers between bilateral ovaries, and the frequency distribution of the number difference in bilateral ovarioles was analyzed.

### 2.4. Effect of Host Species, Host Instars, and Field or Indoor Breeding on Ovariole Number and Body Size

The total ovariole number and body size of *C. chlorideae* was researched in female adult wasps aged 5–10 days under different treatments, respectively. (1) Four noctuid species, *H. armigera*, *M. separata*, *S. litura*, and *S. frugiperda*, were chosen to study the effect of host species. The 2nd instar larvae of each host species were parasitized by *C. chlorideae*. Finally, about 24–40 female wasps reared in each host species were, respectively, measured to determine ovariole number and body size. The body size was determined by measuring the length of the hind tibia under a calibrated stereomicroscope. (2) The 1st, 2nd and 3rd instar larvae of *H. armigera* were parasitized, respectively. About 26–43 female wasps reared, respectively, in different host instars were measured to determine ovariole number and body size. (3) *C. chlorideae* were captured from a tobacco field, and bred indoors in the 2nd instar larvae of *H. armigera* for several generations under adequate food and suitable environment. The ovariole number and body size were compared between female wasps collected in the field and their next generations bred indoors.

### 2.5. Correlation between Ovariole Number and Body Size

A total of 98 female wasps of *C. chlorideae* were, respectively, measured to determine ovariole number and body size for analyzing their correlation. Body size was divided into three ranges, large (>1.5 mm), medium (1.3–1.5 mm), and small (<1.3 mm), and the ovariole numbers of different ranges were statistically analyzed. Further, the correlation between ovariole number and body size was analyzed according to Pearson correlation coefficient, and the correlation was checked depending on *p* value at α = 0.05.

### 2.6. Statistical Analysis

Analysis of variance (ANOVA) followed by Tukey HSD tests were performed to test the significance of differences in ovariole number and body size affected by host species, host instars and field/indoor breeding. The relationship between ovariole number and body size was determined by ANOVA followed by Tukey HSD tests, and the correlation was analyzed by two-tailed Pearson correlation coefficient. The level of significant differences between the means of two data sets was *p* < 0.05, and the extremely significant level was *p* < 0.01. All of the statistical tests were performed by SPSS version 17.0 software.

## 3. Results

### 3.1. Morphological Observation

#### 3.1.1. Morphological Structure of the Female Internal Reproductive System

**Figure 1:**
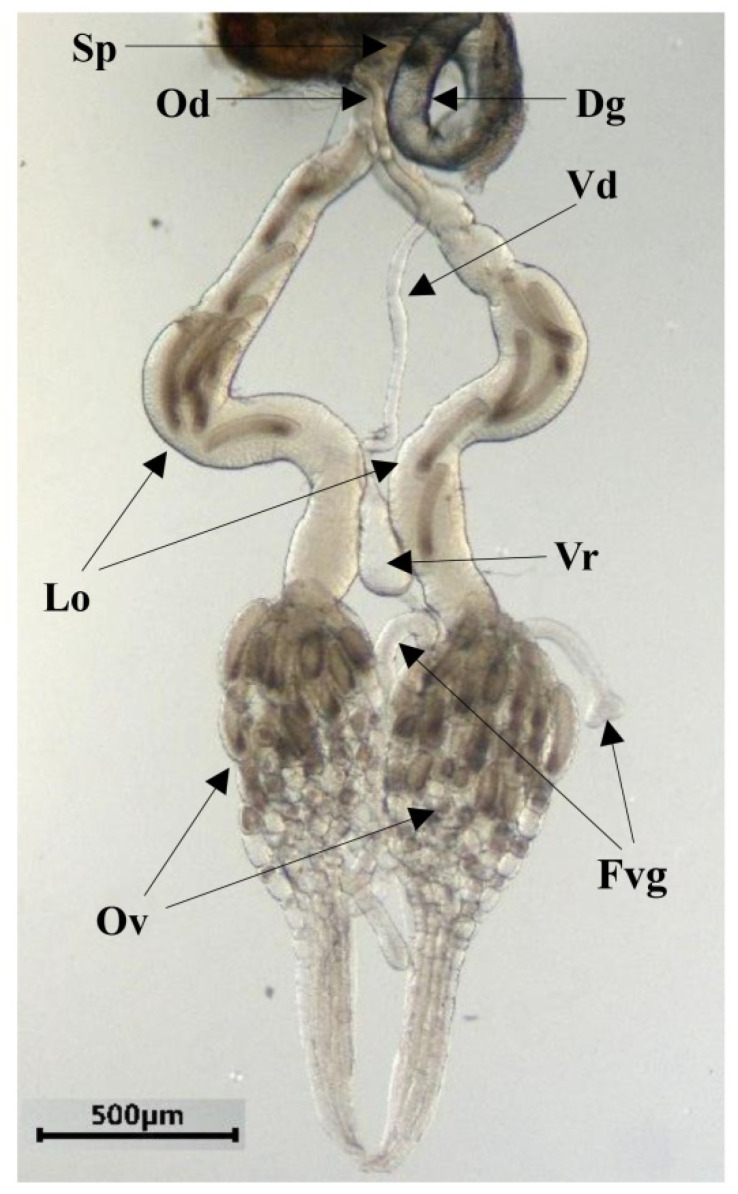
General morphology of internal reproductive system in *C. chlorideae* female. The figure shows the two ovaries (Ov), two lateral oviducts (Lo), a common oviduct (Od), a spermatheca (Sp), a Dufour’s gland (Dg), a venom reservoir (Vr), a venom duct (Vd), and the venom gland composed of two long filaments (Fvg).

The *C. chlorideae* female internal reproductive system is formed by a pair of tadpole-shaped ovaries, a pair of lateral oviducts connected to a common oviduct, a spermatheca, a Dufour’s gland, and a venom apparatus composed of a venom reservoir, venom gland and venom duct (Figure 1). A pair of ovaries are connected to a pair of lateral oviducts, respectively. Each ovary is surrounded by a network of muscle fibers called the peritoneal sheath. Oviducts load mature eggs developed from the paired ovaries. The two lateral oviducts converge, finally, into a common median oviduct which leads to the oviposition apparatus. Each ovary is composed of ovarioles containing the developing oocytes. The front end of each ovariole filament is assembled into a sling, which is attached to the inner surface of the dorsal integument of the second and third abdominal segments.

#### 3.1.2. Morphological Structure of Ovarioles

**Figure 2:**
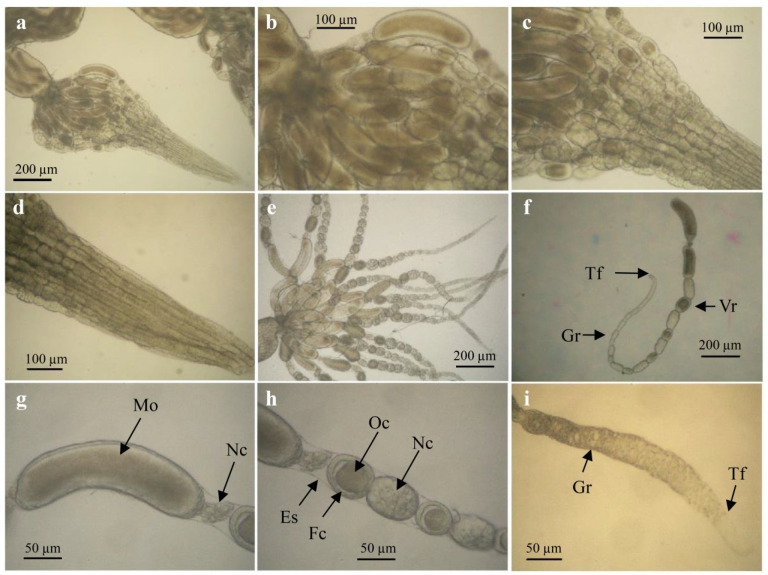
Morphology of ovarioles organization in *C. chlorideae*. (**a**) A series of ovarioles is packaged together in a bundle, in each of the two ovaries. (**b**) The posterior region of a single ovary, with progressively older egg chambers toward the posterior. (**c**) The middle region of a single ovary. (**d**) View of the anterior region of ovary. (**e**) After removing the ovarian peritoneal sheath which envelops the outside of ovary, showing sixteen discrete tubular ovarioles. There are approximately 6-8 egg chambers per ovariole, connected to each other by stalks of follicle cells. (**f**) A single ovariole consists of the germarium (Gr), vitellarium (Vr), and terminal filament (Tf) in three parts. (**g**) View of the ovarian tube base. Signs of programmed cell death in nurse cells (Nc), indicating the mature oocyte (Mo) with a chorion. (**h**) View of the ovariole is surrounded by an epithelial sheath (Es), showing the differentiated oocyte (Oc) surrounded by follicular cells (Fc) and accompanying nurse cells (Nc) within the ovariole. (**i**) View of the anterior region of the ovariole (germarium), with the terminal filament of ovariole showing the structural difference between the typical flat and strongly chromogenic somatic cells and the weak cell type.

Females of *C. chlorideae* displayed ovaries composed by ovarioles of the meroistic polytrophic type. Some discrete tubular ovarioles are packaged together in parallel to form an ovary (Figure 2a–e). Each ovariole mainly consists of germarium, vitellarium, and terminal filament in three parts (Figure 2f). Each ovariole contains a succession of developing egg chambers made up of an oocyte and some nurse cells. New egg chambers arise at the germarium, which is at the apical tip of the ovariole. In the vitellarium, there are approximately 6–8 egg chambers per ovariole connected to each other by stalks of follicular cells (Figure 2f,i). The nurse cells nourish the oocytes during early growth stages, and the follicular cells provide materials for the yolk and make the chorion. In progressively older egg chambers toward the posterior, eggs are mature and accompanied by programmed cell death in nurse cells (Figure 2g,h).

### 3.2. Intraspecific Variation in the Number of Ovarioles

**Figure 3:**
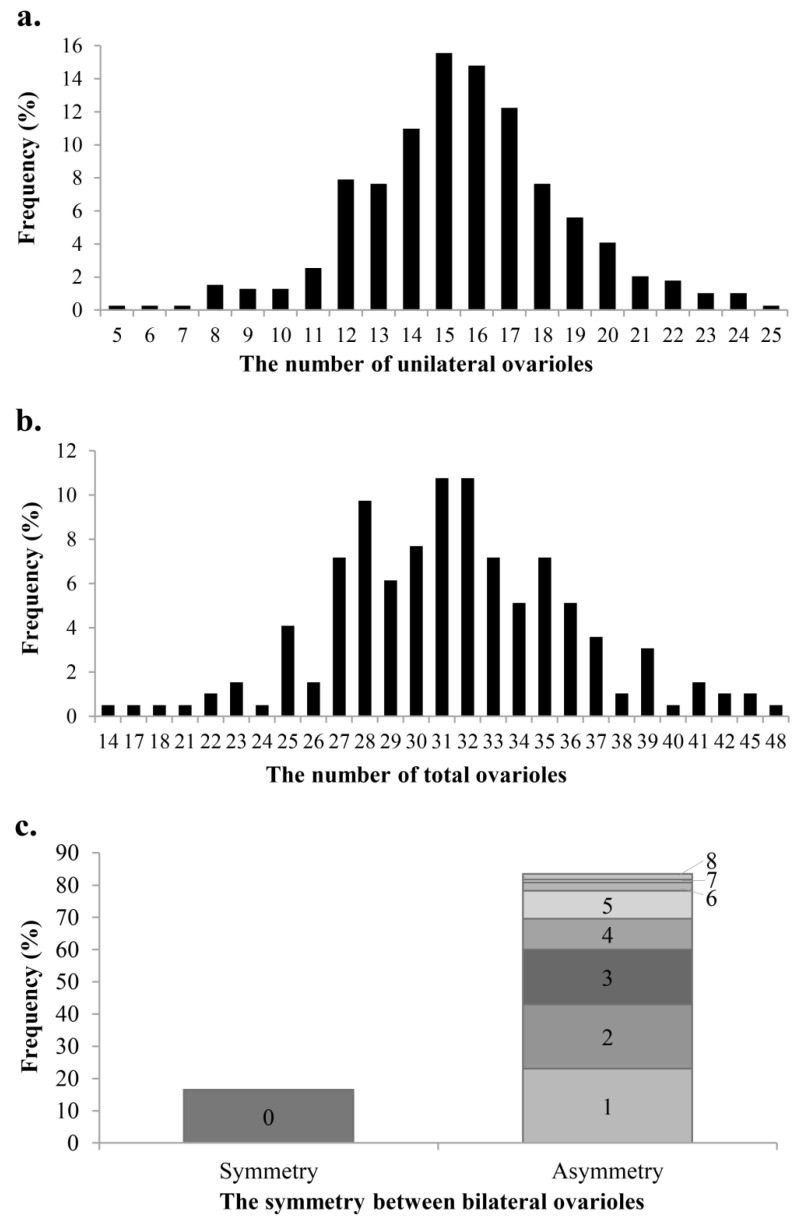
The ovariole numbers in the female of *C. chlorideae*. The frequency of ovariole numbers in unilateral ovary (**a**) and bilateral ovaries (**b**) was counted, respectively. (**c**) The symmetry of ovariole number between bilateral ovaries was analyzed; the number on column bar represents the difference in quantity between bilateral ovarioles (n = 230 adults aged 0–10 days).

The ovariole number of the unilateral ovary varied greatly from 5 to 25, with a five-fold difference; the mean ± SD was 15.6 ± 0.8. The unilateral ovariole number of highest frequency was 15, which accounted for 15.6% of total statistical samples. The ovariole numbers of lowest frequency were five, six, seven, and twenty-five, which all accounted for 0.3%. The sum of the frequency of the unilateral ovary with 14–17 ovarioles was more than half, at 53.6% (Figure 3a).

The number of total ovarioles in bilateral ovaries varied greatly from 14 to 48, with a difference of 3.4 times; the mean ± SD was 31.4 ± 1.1. The number of total ovarioles with highest frequency was 31 and 32, both accounting for 10.8% of the total statistical samples. The ovariole numbers of lowest frequency were 14, 17, 18, 21, 24, 40, and 48, which all accounted for 0.5%. The frequency of 27–36 added up to 76.9% (Figure 3b).

The number of ovarioles may not be equal between bilateral ovaries, even within the same individual (Figure 3c). The difference in quantity between bilateral ovaries varied from 0 to 8. The difference of 0 meant that the number of ovarioles between bilateral ovaries was equal or symmetric, and its frequency was 16.5%. The difference of 1–8 represented that the number of ovarioles between bilateral ovaries was different or asymmetrical, which accounted for 83.5%. The results indicated that the ovariole number of *C. chlorideae* fluctuates over a wide range, and the overall change rule of unilateral and total ovariole numbers was consistent with the normal distribution.

### 3.3. Effect of Host Species on Ovariole Number and Body Size

**Figure 4:**
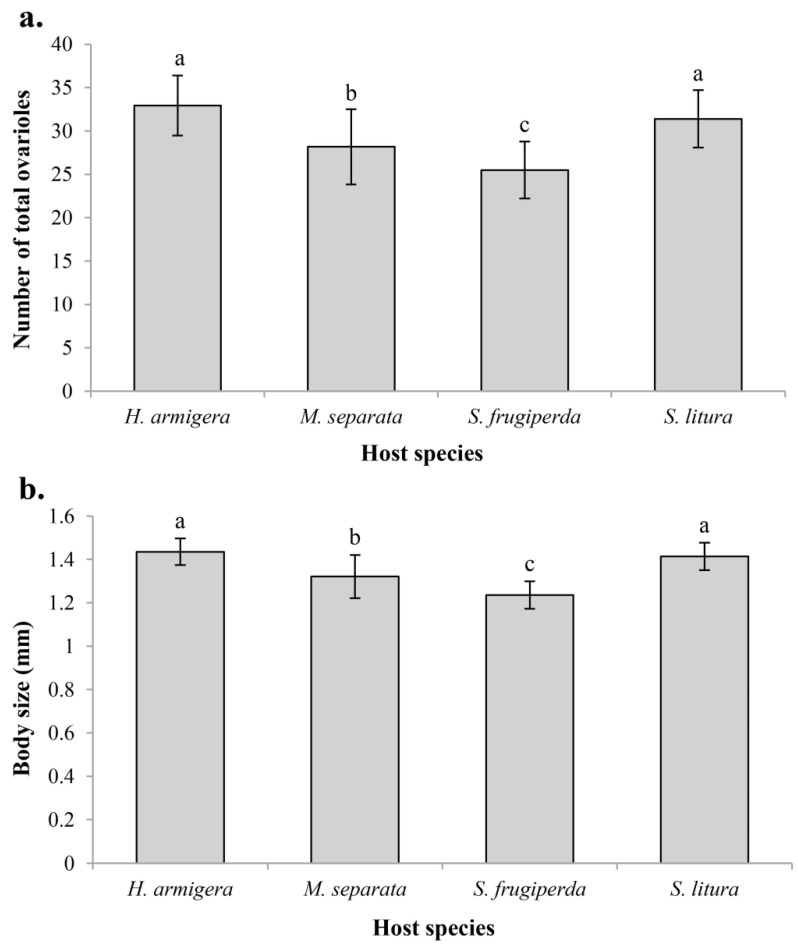
Variation in ovariole number (**a**) and body size (length of hind tibia) (**b**) of *C. chlorideae* depending on four host species. The histogram and error bars show the mean and standard deviation. Different letters above the bars represent that the difference between groups has reached an extremely significant level (*p* < 0.01), and the same letter indicates that there is no significant difference (*p* > 0.05). Statistical analysis was performed using ANOVA and Tukey HSD tests. Sample sizes: n*_H. armigera_* = 40, n*_M. separata_* = 40, n*_S. frugiperda_* = 40, n*_S. litura_* = 40 for ovariole number test; n*_H. armigera_* = 28, n*_M. separata_* = 24, n*_S. frugiperda_* = 26, n*_S. litura_* = 30 for body size test.

Host species have a significant effect on both the ovariole number and body size of *C. chlorideae*. Significant differences were found among the various groups of different host species, not only in ovariole number (d.f. = 3, 156; *F* = 33.899; *p* < 0.001) but also in body size (d.f. = 3, 104; *F* = 43.280; *p* < 0.001) (Figure 4). *C. chlorideae* had the largest number of ovarioles when reared in *H. armigera*. Although the number of ovarioles was greater in *H. armigera* than in *S. litura*, there was no significant difference (*p* = 0.225). The ovariole number of wasps reared in *M. separata* was significantly less than *S. litura*, and wasps reared in *S. frugiperda* had the smallest number of ovarioles. Except for the comparison of *H. armigera* vs. *S. litura*, all other pairwise comparisons reached extremely significant differences (*p* < 0.01) (Figure 4a). Coincidentally, the effect of host species on the body size of *C. chlorideae* had a similar result (Figure 4b). Females of *C. chlorideae* increased their ovariole number by 29.2% and body size by 16.2% when they were reared in *H. armigera* compared to *S. frugiperda*. These results suggest that *H. armigera* is the optimal host of *C. chlorideae*, and *S. litura* can be considered as an excellent alternative host.

### 3.4. Effect of Host Instars on Ovariole Number and Body Size

**Figure 5:**
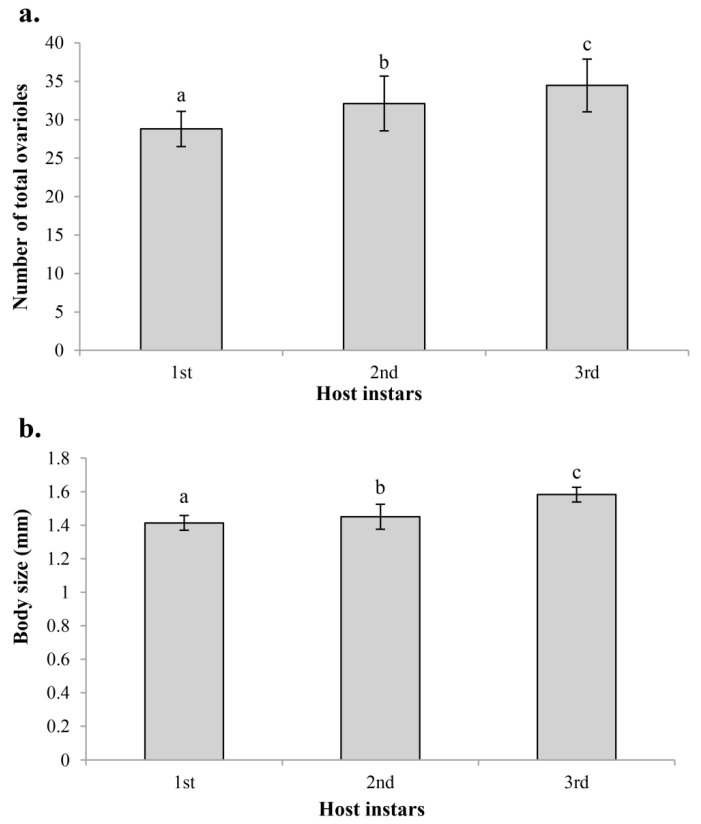
Variation in ovariole number (**a**) and body size (length of hind tibia) (**b**) of *C. chlorideae* depending on the host instars. The histogram and error bars show the mean and standard deviation. Different letters above on the bars represent that the difference between groups has reached a significant level (*p* < 0.05). Statistical analysis was performed using ANOVA and Tukey HSD tests. Sample sizes: n_1st_ = 30, n_2nd_ = 30, n_3rd_ = 30 for ovariole number test; n_1st_ = 41, n_2nd_ = 43, n_3rd_ = 26 for body size test.

Host instars had a significant effect on the ovariole number and body size of *C. chlorideae*. Significant differences were found among the various groups of different host instars, not only in ovariole number (d.f. = 2, 87; *F* = 24.613; *p* < 0.001), but also in body size (d.f. = 2, 107; *F* = 71.655; *p* < 0.001). The ovariole number and body size of female wasps both increased gradually with the increase of host instars (Figure 5). The wasps reared in the third instar hosts had the highest ovariole number, which was 19.7% more than those reared in the first instar hosts (*p* < 0.001) and 7.4% more than those reared in the second instar hosts (*p* = 0.012). The ovariole number of wasps reared in the second instar hosts was also more than that of those reared in the first instar hosts (*p* < 0.001) (Figure 5a). The change in body size of *C. chlorideae* showed a similar trend. The wasps reared in the third instar hosts had the biggest body size, which had an extremely significant result at 12.0% longer than those reared in first instar hosts (*p* < 0.001) and 9.1% longer than those reared in second instar hosts (*p* < 0.001). The body size of wasps reared in second instar hosts was also bigger than those reared in first instar hosts (*p* = 0.012) (Figure 5b). This result indicates that the third instar larva of *H. armigera* is the optimal instar, which provides a high quality growing environment for the development of *C. chlorideae*.

### 3.5. Correlation between Ovariole Number and Body Size

**Figure 6:**
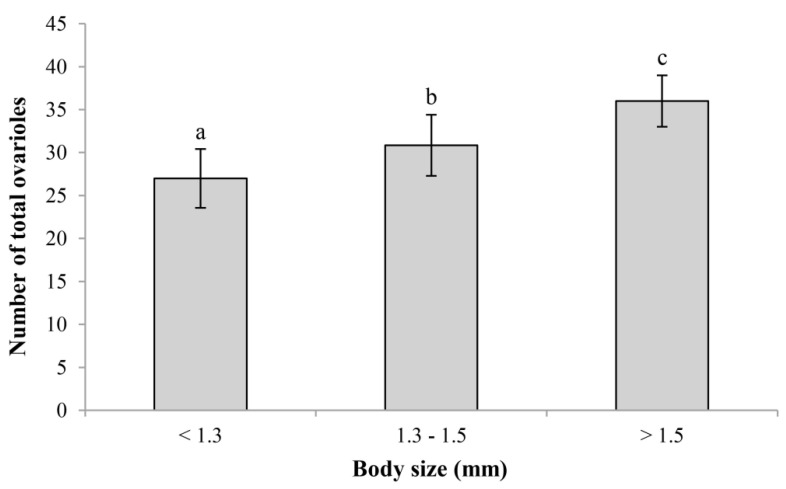
Variation in ovariole number in different body sizes (length of hind tibia) of *C. chlorideae*. The histogram and error bars show the mean and standard deviation. Different letters above the bars represent that the difference between groups has reached an extremely significant level (*p* < 0.01). Statistical analysis was performed using ANOVA and Tukey HSD tests. Sample sizes: n_<1.3_ = 9, n_1.3–1.5_ = 64, n_>1.5_ = 25.

**Figure 7:**
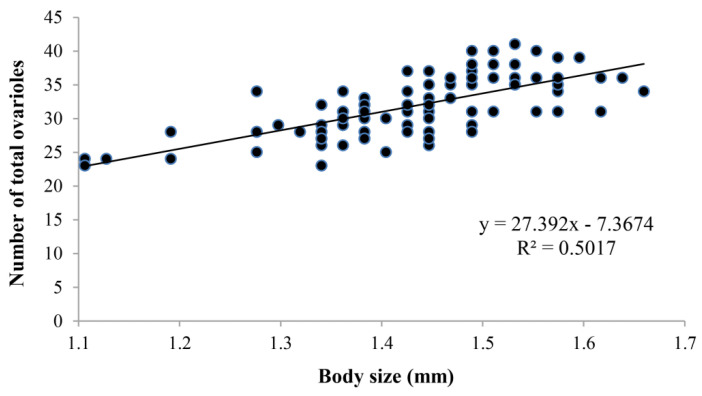
Correlation between ovariole number and body size (length of hind tibia) of *C. chlorideae*. These dots show body size and ovariole number of female individuals of *C. chlorideae*. The body size and corresponding ovariole number was fitted with trend lines, equation and R^2^ (n = 98).

Body size had a significant effect on the ovariole number (d.f. = 2, 95; *F* = 30.347; *p* < 0.001). The ovariole number increased gradually with the increase in body size. Large wasps (body size > 1.5 mm) had more ovarioles than medium wasps (body size in 1.3–1.5 mm, *p* < 0.001) and small wasps (body size < 1.3 mm, *p* < 0.001) (Figure 6). There was a significant positive correlation between ovariole number and body size (n = 98, r^2^ = 0.501, *p* < 0.001) (Figure 7), where a larger body size represented more ovarioles. 

### 3.6. Effect of Artificial Breeding on Ovariole Number and Body Size

**Figure 8:**
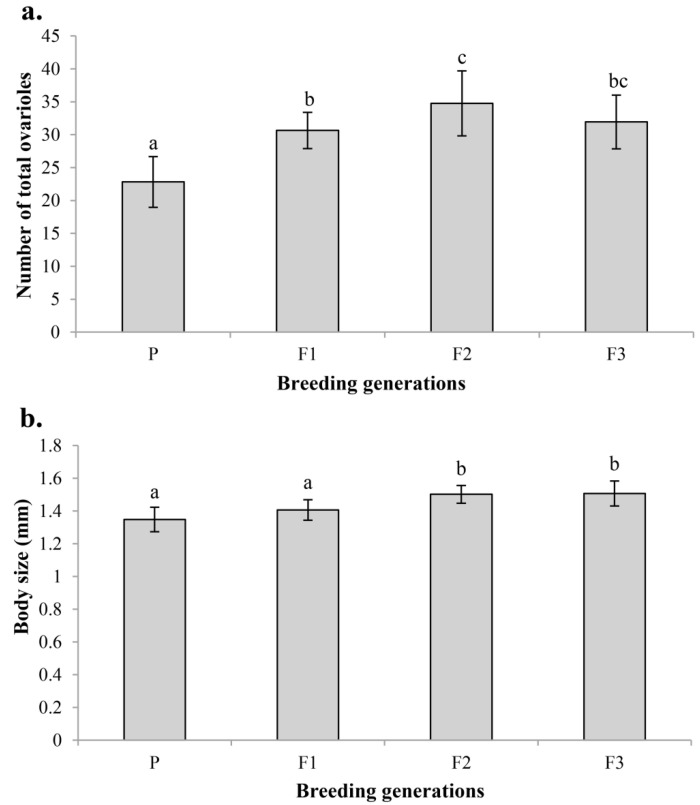
Variation in ovariole number (**a**) and body size (length of hind tibia) (**b**) of *C. chlorideae* after several breeding generations. The histogram and error bars show the mean and standard deviation. P: parental generation captured in the fields, F_1_: the first breeding generation, F_2_: the second breeding generation, F_3_: the third breeding generation. Different letters above on the bars represent that the difference has reached an extremely significant level (*p* < 0.01), and the same letter indicates that there is no significant difference (*p* > 0.05). Statistical analysis was performed using ANOVA and Tukey HSD tests. Sample sizes: n_p_ = 11, n_F1_ = 23, n_F2_ = 12, n_F3_ = 17 for ovariole number and body size test.

Optimized artificial breeding could improve both the ovariole number (d.f. = 3, 59; *F* = 20.802; *p* < 0.001) and the body size (d.f. = 3, 59; *F* = 17.687; *p* < 0.001) (Figure 8). The ovariole number of the parental generation bred naturally in the wild was significantly lower compared with later generations (F_1_, F_2_ and F_3_) bred artificially under laboratory conditions (*p* < 0.001). The F_2_ generation had the most ovarioles, which was significantly more than F_1_ (*p* = 0.019) and numerically more than F_3_ (*p* = 0.215) (Figure 8a). The body size showed similar changes. The body size of the parental generation bred naturally in the wild was the smallest, and numerically smaller than F_1_ generation (*p* < 0.099), and was extremely significantly smaller than F_2_ and F_3_ generation (*p* < 0.001). Body size was almost equal between F_2_ and F_3_ (*p* = 0.997) (Figure 8b).

## 4. Discussion

The paired ovaries of *C. chlorideae* consist of polytrophic ovarioles. The total ovariole number of *C. chlorideae* can reach up to 48, which is more than that of *V. canescens*, up to 26, although the latter has a bigger body size [13]. The ovariole number shows species-specificity, which was dominated by the genetics of the species. Moreover, the number of ovarioles was asymmetric in bilateral ovaries for most females of *C. chlorideae*, which accounts for 83.5% (Figure 3c). Moreover, the ovariole number of *C. chlorideae* showed great intraspecific variation, and the unilateral ovariole number (from 5 to 25) and total ovariole number (from 14 to 48) both varied widely (Figure 3a,b), which indicated that the ovariole number of female wasps can be affected by environmental factors. Intraspecific differences in ovariole number can occur based on genetic and stressing factors [25]. The number of ovarioles could be affected by food, temperature, intra-species hierarchy, and so on [26,27].

Host species is one of the important factors influencing the development of the parasitoids. Our study showed that *C. chlorideae* had the largest number of ovarioles and body size when they were reared in *H. armigera* rather than other test host species (Figure 4). Another study also showed that the larvae of *C. chlorideae* developed better in *H. armigera* than *M. separata* and *S. exigua*, and those individuals developing in *H. armigera* had higher percentage of unencapsulated eggs and heavier cocoons [5]. Depending on the studies of host suitability, *H. armigera* was considered to be the most suitable host for *C. chlorideae*. Host adaptation is related to the ability to adjust to the host immune response, and *C. chlorideae* shows a stronger adjustment in its suitable host by decreasing phenoloxidase activity and immune hemocyte number [5]. In addition, we believe that the difference in ovariole number or body size of *C. chlorideae* may be caused by the different nutrients access during the embryonic and larval development of parasitoid in different host species. Nevertheless, *H. armigera* and *S. frugiperda* are highly cannibalistic and therefore uneconomical for mass rearing *C. chlorideae* [28,29,30]. *M. separata* and *S. litura* can be used as alternative hosts due to having little or no cannibalism [30,31].

Moreover, ovariole number and body size could also be affected by host larval instars. The female wasps of *C. chlorideae* had more ovarioles and a bigger body size reared in the third instar host than those in the first or second instar hosts (Figure 5). However, there was a higher probability that the eggs were encapsulated when female wasps laid eggs in the fourth instar or older hosts. *Dolichogenidea gelechiidivoris* Marsh (Hymenoptera: Braconidae) also showed that female wasps had significantly fewer eggs when they were reared in young instar host (first) than old instar hosts (third and fourth) [32], which was similar to our result that wasps reared in young instar hosts (first) would have fewer ovarioles, which represented less eggs. Although young host larvae have weaker immune defenses, they also have less nutrients available to the developing parasitoid. However, the old host larvae usually have a stronger immune defense to encapsulate and kill the parasitoid. Therefore, a suitable instar of the host is also important; the third instar larva of *H. armigera* was considered the most suitable instar for breeding *C. chlorideae*.

According to our results, there was a positive correlation between body size and ovariole number. The correlation also applied to other parasitoids, such as *Dastarcus helophoroides* Fairmaire (Coleoptera: Bothrideridae) (R^2^ = 0.9704, *p* < 0.001, n = 16) [33] and *V. canescens* (R^2^ = 0.7851, *p* < 0.001, n = 50) [13], which was stronger in the two species than *C. chlorideae* (R^2^ = 0.5017, *p* < 0.001, n = 98). Larger wasps had significantly more eggs and ovarioles than smaller wasps [13]. The result suggests that the bigger body size supports females to develop more ovarioles, and the body size can be as an indicator of ovariole number. Thus, a breeding procedure to develop large female adults might be crucial for successful biological control.

These conclusions suggest that it is important to create favorable artificial breeding conditions for improving the quality of *C. chlorideae*. Some factors, such as suitable host species, host instars, breeding environment (temperature, illumination, humidity, etc.) and food supply, are important for improving the rate of successful parasitism, body size, ovariole number, egg loads, lifespan, vitality and fecundity. By optimizing the artificial indoor breeding conditions, the body size and ovariole number of *C. chlorideae* have both been significantly improved compared with wild wasps (Figure 8). However, long-term continuous artificial rearing could lead to deterioration of the *C. chlorideae* colony unless wild individuals are introduced into the colony [29]. Further research is necessary to determine the effects of rearing duration on colony collapse in *C. chlorideae*. 

Our works revealed the ovariole morphology and the effect of the rearing host on the ovariole number and body size of *C. chlorideae*. The ovariole number and body size have positive impacts on the biocontrol capacity of natural enemies [22,34]. Based on our research, we consider that both body size and ovariole number can be used as indicators to evaluate the quality of *C. chlorideae*, and the difference in the two indicators can represent the difference in fecundity or biocontrol efficacy. This study is of great significance for providing a good foundation for improving laboratory mass breeding and other important assays of *C. chlorideae*.

## Data Availability

Not applicable.
